# Validation of the Polish version of the Western Ontario Rotator Cuff Index in patients following arthroscopic rotator cuff repair

**DOI:** 10.1186/s12891-018-2238-9

**Published:** 2018-09-12

**Authors:** Agnieszka Bejer, Mirosław Probachta, Marek Kulczyk, Sharon Griffin, Elżbieta Domka-Jopek, Jędrzej Płocki

**Affiliations:** 1 Institute of Physiotherapy, Faculty of Medicine, University of Rzeszow, ul. Hoffmanowej 25, 35-016 Rzeszow, Poland; 2 The Holy Family Specialist Hospital, Rudna Mała, Poland; 3 Fowler Kennedy Sport Medicine Clinic, University of Western Ontario, London, Canada; 4 Faculty of Medicine, University of Information Technology and Management, Rzeszow, Poland

**Keywords:** WORC, Rotator cuff tear, Quality of life questionnaires, Validity, Reliability

## Abstract

**Background:**

The Western Ontario Rotator Cuff Index (WORC) is a joint specific outcome tool that assesses the quality of life in patients with various rotator cuff problems.

Our purpose was to evaluate selected psychometric characteristics (internal consistency, validity, reliability and agreement) of the Polish version of WORC in patients undergoing rotator cuff repair.

**Methods:**

Sixty-nine subjects took part in the study with a mean age 55.5 (range 40–65). All had undergone arthroscopic rotator cuff repair in 2015–2016. Data from 57 patients in whom symptoms in the shoulder joint had not changed within 10–14 days were analyzed in a WORC test-retest using the Intraclass Correlation Coefficient (ICC), Standard Error of Measurement (SEM) and Minimal Detectable Change (MDC). WORC was compared to the short version of the Disabilities of Arm, Shoulder and Hand Questionnaire (QuickDash) and the Short Form-36 v. 2.0 (SF-36).

**Results:**

High internal consistency of 0.94 was found using Cronbach’s alpha coefficient. Reliability of the WORC resulted in ICC = 0.99, agreement assessed with SEM and MDC amounted to 1.62 and 4.48 respectively. The validity analysis of WORC showed strong correlations with QuickDash and SF-36 PCS (Physical Component Summary), while moderate with SF-36 MCS (Mental Component Summary). WORC had no floor or ceiling effect.

**Conclusions:**

The Polish version of the WORC is a reliable and valid tool with high internal consistency for assessing the quality of life in patients undergoing arthroscopic rotator cuff repair.

## Background

Rotator cuff disorders are one of the most common shoulder problems which can significantly affect a patients’ ability to work and other activities of daily life such as dressing, washing hair, driving etc. [1, 2]. Little is known about the incidence of it or associations of its demographic in the general population both in Poland and worldwide. White et al. conducted a large epidemiological study of rotator cuff pathology in the United Kingdom. They observed that the incidence of rotator cuff pathology was 87 per 100,000 person-years. At the same time, it was more common in women (90 cases per 100,000 person-years) than in men (83 cases per 100,000 person-years). They found the highest incidence of 198 per 100,000 person-years in those aged between 55 and 59 years [3]. Mall et al. conducted a systematical review of the literature in 2013 and found that the injury usually causes damage to the tendon of supraspinatus in 84% of tears, infraspinatus in 39% of cases, and the tendon of subscapularis was damaged in 78% of injuries. They also observed that the tear size of the tendon was < 3 cm in 22%, 3 to 5 cm in 36%, and > 5 cm in 42% [4]. In the systematical review Littlewood et al. found the incidence of rotator cuff tendinopathy to range from 0.3 to 5.5% and annual prevalence from 0.5 to 7.4%. [5].

A large number of measurement tools for evaluating patients with shoulder problems are available for assessing symptoms, functioning and quality of life, the majority of which were developed in English-speaking countries. Some of those questionnaires available are: the Western Ontario Shoulder Instability Index (WOSI) [6] and the Shoulder Instability Questionnaire (SIQ) [7], the Western Ontario Osteoarthritis of the Shoulder Index (WOOS) [8], the Western Ontario Rotator Cuff Index (WORC) [9] and the Rotator Cuff Quality of Life Index (RC-QOL) [10], the Disabilities of the Arm, Shoulder and Hand Questionnaire (DASH) [11] and its shortened version - QuickDASH [12, 13].

Before being introduced into scientific research and / or clinical practice, outcome tools that were created in a country of a different language or culture need to be adapted. Adaption consists of two phases: a linguistic validation and a psychometric validation and should be conducted according to specific methodology [14–16].

The authors of this paper undertook to adapt and validate the WORC to the Polish version, because up to now there has been no published results of psychometric analysis of the Polish version of questionnaires used to assess the quality of life of patients with rotator cuff disorders. The Western Ontario Rotator Cuff Index is a widely used outcome tool for assessing the quality of life in patients with various problems in the rotator cuff. The analysis of the results of the questionnaire allows for a reliable assessment of the symptoms and functioning (5 domains) of such a patient and verification of the effectiveness of the treatment applied. The questionnaire was originally created in English and published by Kirkley et al. in 2003, who confirmed its high reliability, accuracy and sensitivity [9]. Also, in a systematic review on the patient-reported outcomes used for the evaluation of symptoms and functional limitations in people with rotator cuff problems, St-Pierre et al. concluded that the WORC is one of the most responsive questionnaires and showed good psychometric properties for the targeted population [17].

The WORC questionnaire has been translated into several languages including German [18], Turkish [19], Portuguese for use in Brazil [20], Norwegian [21], Iranian [22], Dutch [23], Japanese [24], Swedish [25] and Chinese [26].

Cultural-linguistic adaptation of the WORC questionnaire to the Polish version was carried out in 2017 by Bejer et al. [27] in accordance with the Mapi Research Institute guidelines [14]. It is proceeded by six stages: preparation of two independent versions of the translation of the WORC questionnaire from English into Polish, reconciliation of the common version, preparation of a back translation into English, comparison of the back translation with the source version of the questionnaire by the original author and corrections, verification of the received version of the questionnaire by a team of experts, testing the questionnaire in a group of patients diagnosed with rotator cuff injury and making corrections - obtaining the Polish version of WORC-PL [27]. Each stage of linguistic adaptation was completed with a written report sent to the author of the source version.

The purpose of this study was to evaluate selected psychometric properties (internal consistency, reliability and agreement, validity) of the Polish version of the WORC questionnaire.

## Methods

Patients were identified from the hospital patient database of the Orthopedic Department in the Specialist Hospital in Rudna Mała, Poland. Patients who had undergone arthroscopic reconstruction of the rotator cuff from 2015 to 2016 by one orthopedic surgeon (supraspinatous, infraspinatous or subscapularis, and additionally after the damage to the tendon of the long head of the biceps brachi), were eligible for the study.

Patients who were ≥ 18 years of age, were native Polish speakers and signed an informed consent participated in the study. Exclusion criteria was: previous surgeries within the shoulder complex and the upper limb, dislocations of the shoulder complex, fractures of the shoulder blade, clavicle or upper limb, and who had co-existing rheumatological or neurological conditions.

### Outcome Tools

#### The Western Ontario Rotator Cuff Index (WORC)

The WORC is a self-reported questionnaire, which contains 21 items grouped into five domains – physical symptoms (six items), sport/recreation (four items), work (four items), lifestyle (four items) and emotions (three items). It also includes instructions for patients on how to complete the questionnaire and in case of problems in fully understanding the questions there are detailed explanations for each. The patients give responses concerning problems and symptoms they have observed over the past week by placing a slash “/” on a 10 cm (100 mm) visual-analogue scale. In order to calculate the result, the distance from the left side of the line is measured and the result calculated out of 100 (with the accuracy of 0.5 mm). The total score for each domain can be calculated (Physical symptoms / 600; Sport and Rrecreation / 400; Work / 400; Lifestyle / 400; Emotions / 300). The best possible result in the whole questionnaire is 0, the worst is 2100. The result presented in a clinically meaningful way is the percentage of the basic result. Since the worst possible result is 2100, the total score is subtracted from 2100, and then divided by 2100 and multiplied by 100, to obtain a percentage. WORC score can therefore vary from 0% - ie the lowest level of functional status, up to 100%, ie the highest level of functioning [9, 27].

#### The Short Form-36 v. 2.0 (SF-36 v. 2.0)

The SF-36 v. 2.0 is a generic health related quality of life (HRQOL) questionnaire with 36 questions grouped in eight dimensions: physical functioning, role limitations due to physical health, role limitations due to emotional health, bodily pain, vitality, social functioning, general health and mental health. The score for each domain can range from 0 to 100, the lower the score, the worse the quality of life [28]. From these 8 dimensions, 2 summary scores, 1 for physical health (PCS - Physical Component Summary) and 1 for mental health (MCS - Mental Component Summary), can be computed [29, 30].

#### The abridged version of the Disabilities of Arm, Shoulder and Hand Questionnaire (QuickDash)

The QuickDash is a self-completed instrument for assessment of disability of the upper limb, viewed as a functional whole. It contains 11 questions: symptoms (3 questions) and the impact of upper limb problems on social activity, limitations at work or everyday activities (8 questions). The response format is a five-point Likert scale, where the lowest value means no restrictions or absence of the symptom, and the highest - lack of possibility to perform the activity or maximum severity of the symptom. The QuickDASH constraint and symptom index is calculated by: summing the circled digits, dividing by the number of responses, subtracting 1 and multiplying by 25. The index takes the form of a number between 0 and 100, where a higher value means a greater limitation in performing activities [12, 13].

### Procedure

The subjects were tested two times. The first test consisted of completing the Polish versions of all questionnaires: WORC, QuickDash and the Short Form-36 (SF-36) v. 2.0. The second test was performed 1–2 weeks later. People answered the WORC questionnaire (re-test) and reported whether their symptoms had changed on a 7-point Global Rating of Change Scale (GRC) (1 = much better, 2 = somewhat better, 3 = a little better, 4 = no change, 5 = a little worse, 6 = somewhat worse, 7 = much worse) [31].

The test-retest time interval was used according to the scientific literature, which indicates that an interval of 1–2 weeks is adequate and reasonable in such studies. There is little probability of changes in symptoms during such a period and time is long enough for the respondents to forget their previous answers [32, 33].

### Statistical analysis

All analyses were conducted using the Statistica 10.0 software. The level of statistical significance was assumed a priori at α < 0.05. Normal distribution of the results of this study was verified using Shapiro-Wilk test. After determining that the data has a non-normal distribution, the non-parametric Wilcoxon test for the basic statistical analysis was used. The dependencies between total and domain scores of the WORC and between total WORC and reference measures (SF-36, QuickDASH) were determined using the non-parametric Spearman’s rank correlation coefficient. The sample size was based on the general recommendations of Altman of at least 50 subjects in a methods comparison study [34].

#### Internal consistency

Internal consistency is a measure of the extent to which items in a questionnaire domain are correlated (homogeneous), thus measuring the same concept. It was calculated by using Cronbach’s alpha coefficient and was based on data from a group of 69 patients. The scale shows good internal consistency if value of Cronbach’s alpha is between 0.70 and 0.95 [32, 33].

#### Reliability and agreement

Reliability and agreement concern the degree to which repeated measurements in stable persons (test-retest) provide similar answers. Reliability is the degree to which patients can be distinguished from each other, despite measurement error. Agreement is the absolute measurement error, i.e., how close the scores on repeated measures are. Small measurement error is required for evaluative purposes in which one wants to distinguish clinically important changes from measurement error [33].

The intra class correlation (ICC), with a 95% confidence interval (CI) was used to assess the reliability of the WORC. It was calculated on a group of 57 people who completed the WORC questionnaire two times. According to the guidelines from the literature, we assumed positive rating for reliability when the ICC is ≥ 0.70 [33].

To assess the agreement, we calculated the Standard Error of Measurement (SEM) and the Minimal Detectable Change (MDC) in the group 57 people who completed WORC two times. SEM was calculated using the formula: SEM = SD √(1-R), where SD represents Standard Deviation of the sample and R the reliability parameter (ICC). The MDC is the minimum amount of change in a patient’s score that ensures the change is not the result of measurement error. MDC was calculated using the formula. MDC=SEMx1.96× √2, where 1.96 derives from the 0.95% CI of no change, and √2 shows two measurements assessing the change [33, 35].

#### Construct validity

Construct validity refers to the extent to which scores on a particular instrument relate to other measures in a manner that is consistent with theoretically derived hypotheses concerning the concepts that are being measured [33].

To evaluate the construct validity of the Polish version of the WORC, the Spearman’s correlation coefficient (SCC) was calculated between total and domain scores of the WORC and a general quality of life questionnaire (SF-36) and the joint-specific questionnaire to assess the functioning of the upper limb (QuickDASH). Correlation coefficients, *r* < 0.30 = low, 0.30 < *r* < 0.70 = moderate, *r* > 0.70 = high, were used to assess validity [36]. We hypothesized that correlations between the WORC and the QuickDASH would be stronger than between the WORC and the SF-36. We also expected that the WORC total would be stronger correlated with SF - 36 PCS than MCS, while emotions domain of WORC would be stronger correlated with SF-36 MCS than with PCS and QuickDASH.

#### Content validity

Floor or ceiling effects are considered to be present if more than 15% of the respondents achieved the lowest or highest possible score, respectively [33]. Floor and ceiling effects were calculated in the group of 69 patients for WORC, SF-36 PCS, SF-36 MCS and QuickDASH.

## Results

### Participant characteristics

After screening of the database of Specialist Hospital in Rudna Mała we identified 140 patients who were operated on for rotator cuff disorders in 2015–2016 (72 people in 2015 and 68 people in 2016) of which 111 met the inclusion criteria. These patients were contacted by phone, the study explained to them and were asked for their willingness to complete questionnaires. Those that agreed signed an informed consent form and the outcome tools were sent by post according to the measurement protocol. Sixty-nine subjects (62.2%) returned the completed questionnaires. Patient gender, age, operated limb, hand dominance, time from surgery and diagnosis were recorded (Table 1).

**Table 1:** Patient demographic and clinical characteristics

	*N* (%)	$\overline{x}$ (range)
Gender		
Male	49 (71)	
Female	20 (29)	
Age (years)		55.5 (40–65)
Handedness		
Right-handed	58 (84)	
Left-handed	11 (16)	
Time from operation		16.8 mths (6–24)
Diagnosis – Tendon injury in:		
4 muscles:	7 (10.1)	
SST + IST + SScapT+LHBT	7 (100.0)	
3 muscles:	22 (31.9)	
SST + SScapT+LHBT	7 (31.8)	
SST + IST + LHBT	11 (50.0)	
IST + SScapT+LHBT	2 (9.1)	
SST + IST + SScapT	2 (9.1)	
2 muscles:	24 (34.8)	
SST + LHBT	15 (62.5)	
SScapT+LHBT	4 (16.7)	
SST + SScapT	5 (20,8)	
1 muscle:	16 (23.2)	
SST	15 (93.7)	
SScapT	1 (6.3)	

*Abbreviations*: *N* number, $\overline{x}$ Mean, *%* per cent, *SST* Supraspinatus Tendon, *IST* Infraspinatus Tendon, *SScapT* Subscapularis Tendon, *LHBT* Long Head Biceps Tendon

In order to assess test-retest reliability and agreement of the scale, the respondents filled in the WORC questionnaire a second time. The median time between administrations was 13 days (range 10–14 days). In the test-retest analyzes, data from 57 people were used; 4 subjects who participated in the first test were excluded due to reporting changes in the functioning of the operated shoulder joint during the assessed period in GRS scale, and 8 people did not return the completed questionnaire for the second time. The absolute values of all scores are given in Table 2.

**Table 2:** Absolute values of all scores

Questionnaire	Total group (*N* = 69)		Group 1 (*N* = 26)		Group 2 (*N* = 43)	
	$\overline{x}$±SD	Range	$\overline{x}$±SD	Range	$\overline{x}$±SD	Range
WORC						
Physical symptoms	77.7 ± 20.4	33.5–100.0	56,1 ± 13,7	33,5-81,0	90,8 ± 9,9	62,5-100,0
Sports/recreation	63.8 ± 29.2	7.8–100.0	32,8 ± 15,6	7,8-75,5	82,6 ± 16,6	43,0-100,0
Work	61.5 ± 31.3	0.0–100.0	29,9 ± 15,5	6,5-62,5	80,6 ± 21,3	0,0-100,0
Lifestyle	73.3 ± 26.0	18.8–100.0	46,4 ± 20,0	18,8-100,0	89,5 ± 11,9	62,3-100,0
Emotions	75.8 ± 24.7	18.0–100.0	52,6 ± 21,5	18,0-92,3	89,8 ± 13,3	43,3-100,0
Total	70.9 ± 24.5	24.0–100.0	44,3 ± 13,6	24,0-76,8	86,9 ± 12,7	50,2-100,0
SF-36						
Physical Functioning	77.5 ± 19.5	15.0–100.0	61,5 ± 19,4	15,0-90,0	87,2 ± 11,8	60,0-100,0
Role Physical	58.8 ± 26.3	6.3–100.0	37,0 ± 15,5	6,3-75,0	71,9 ± 22,4	12,5-100,0
Body Pain	64.9 ± 25.2	22.5–100.0	42,0 ± 16,1	22,5-77,5	78,8 ± 18,8	42,5-100,0
General Health	62.0 ± 13.9	25.0–90.0	57,5 ± 13,9	25,0-80,0	64,8 ± 13,4	40,0-90,0
Vitality	63.0 ± 18.2	18.8–100.0	52,4 ± 14,5	18,8-75,0	69,5 ± 17,3	25,0-100,0
Social Functioning	76.3 ± 22.5	25.0–100.0	61,1 ± 21,0	25,0-100,0	85,5 ± 18,1	37,5-100,0
Role Emotional	75.0 ± 24.8	25.0–100.0	57,4 ± 23,4	25,0-100,0	85,7 ± 19,1	41,7-100,0
Mental Health	71.8 ± 17.9	20.0–100.0	61,5 ± 17,9	20,0-85,0	78,0 ± 15,0	40,0-100,0
PCS	63.3 ± 14.7	26.0–85.7	50,5 ± 12,4	26,0-82,4	71,1 ± 9,8	49,5-85,7
MCS	70.6 ± 18.2	21.4–100.0	58,0 ± 16,9	21,4-83,9	78,3 ± 14,5	48,2-100,0
QuickDash	28.6 ± 24.7	0.0–84.1	52,8 ± 19,2	15,9-84,1	14,0 ± 13,6	0,0-50,0

Group 1 - subject evaluated in the period from 6 months to 1 year after the operation, Group 2 - subjects assessed in from over 1 year to 2 years after surgery

*Abbreviations*: *WORC* Western Ontario Rotator Cuff index, *SF-36 PCS* SF-36 questionnaire Physical Component Summary, *SF-36 MCS* SF-36 questionnaire Mental Component Summary, *QuickDash* an abridged version of the Disabilities of the Arm, Shoulder and Hand questionnaire, $\overline{x}$ Mean, *SD* Standard Deviation

### Internal consistency

Internal consistency was calculated based on the data from test 1. A high degree of internal consistency (α = 0.94) was demonstrated for WORC Total, with an item range of 0.88 to 0.95 and the domain range of 0.92 to 0.95 (Table 3).

**Table 3:** Cronbach’s alpha of WORC calculated for the total score and every domain

WORC	No of items	Cronbach’s alpha
Physical symptoms	6	0.931
Sports/recreation	4	0.945
Work	4	0.940
Lifestyle	4	0.915
Emotions	3	0.922
Total	21	0.941

*Abbreviations*: *WORC* Western Ontario Rotator Cuff index

Correlations between particular detailed measures and between specific measures and a summary measure were shown using the Spearman’s rank correlation coefficient (SCC). All domains are very closely related (from 0.78 to 0.97), as well as very pronounced correlations occur between the domains and WORC Total (from 0.87 to 0.97) (Table 4).

**Table 4:** Spearman’s correlation coefficient (SCC)

WORC	Physical symptoms	Sports/ recreation	Work	Lifestyle	Emotions	Total
Physical symptoms	1	0.94	0.95	0.90	0.79	0.97
Sports/recreation	0.94	1	0.94	0.87	0.82	0.97
Work	0.95	0.94	1	0.91	0.78	0.97
Lifestyle	0.90	0.87	0.91	1	0.88	0.94
Emotions	0.79	0.82	0.78	0.88	1	0.87
Total	0.97	0.97	0.97	0.94	0.87	1

Spearman correlation coefficients showing the strength of the correlations between individual domains of WORC as well as domains and total score

*Abbreviations*: *WORC* Western Ontario Rotator Cuff index

All correlations were significant at *p* < 0.001

### Reliability

#### Intraclass correlation

The results for the WORC scale were compared in test 1 and test 2 (re-test) for a group of 57 patients. In the domain of Work and Lifestyle there were statistically significant differences between the studies, however in the test 2 the results were slightly higher on average by 0.5 points and 1.2 points respectively. The difference is small in relation to the initial result, which in the domain of Work was 61.5 points, and in the domain of Lifestyle was 73.3 points. The ICC for the WORC Total was 0.99, and the domains ranged between 0.94 and 0.99 suggesting a high consistency of the results of both tests for all WORC domains (Table 5).

**Table 5:** Reliability of WORC expressed as ICC calculated for WORC total scores and for the individual domains of WORC. Agreement of WORC expressed as Standard Error of Measurement and Minimal Detectable Change of the WORC

WORC	Change test 2 vs. test 1			*p*	ICC (95% CI)	SEM	MDC
	$\overline{x}$	Me	SD				
Physical symptoms	0.6	0.0	3.2	0.4162	0.9871 (0.9751–0.9934)	2.28	6.32
Sports/recreation	0.4	0.3	2.7	0.1229	0.9960 (0.9923–0.9980)	1.89	5.25
Work	0.5	0.5	4.5	0.0167*	0.9903 (0.9813–0.9950)	3.17	8.78
Lifestyle	1.2	0.3	2.7	0.0209*	0.9935 (0.9855–0.9969)	2.10	5.83
Emotions	−1.4	0.0	8.2	0.4321	0.9491 (0.9041–0.9734)	5.65	15.66
Total	0.4	0.1	2.3	0.1824	0.9955 (0.9913–0.9977)	1.62	4.48

*Abbreviations*: *WORC* Western Ontario Rotator Cuff index, $\overline{x}$ mean, *Me* median, *SD* standard deviation, *SEM* Standard Error of Measurement, *MDC* Minimal Detectable Change, *CI* Confidence Interval

All correlations weren’t significant, except marked * *p* < 0.05

### Agreement

#### Measurement error and minimal detectable change

The standard error of measurement (SEM) associated with the WORC Total was 1.62 points for measurements of a group of 57 subjects, in two subsequent tests. Minimal detectable change MDC for WORC Total was 4.48 points (95% CI). SEM for the individual domains ranged from 1.62 to 5.65 whereas MDC ranged from 4.48 to 15.66. Based on the above results, it can be concluded that change of state has not occurred in the studied group (Table 5).

### Validity

#### Construct validity

Table 6 shows correlations using the Spearman correlation coefficient (SCC), between WORC and reference questionnaires, i.e. SF-36 (results in the domains and in PCS - Physical Component Summary and MCS - Mental Component Summary) and QuickDASH. As expected the WORC Total correlates strongly with SF-36 PCS and moderately with SF-36 MCS. Physical subscales of WORC are more strongly correlated with physical subscales of SF-36. There are strong correlations between WORC domain of Emotions and SF-36 MCS and the correlations with PCS are moderate. However, all correlations between the overall score and the WORC domains and the QuickDash are strong.

**Table 6:** Correlations between WORC (results in domains and overall result) and reference questionnaires

Questionnaire	WORC					
	Physical symptoms	Sports/ recreation	Work	Lifestyle	Emotions	Total
SF-36						
Physical Functioning	0.75^***^	0.71^***^	0.74^***^	0.77^***^	0.61^***^	0.76^***^
Role Physical	0.79^***^	0.75^***^	0.78^***^	0.78^***^	0.65^***^	0.80^***^
Body Pain	0.83^***^	0.82^***^	0.79^***^	0.84^***^	0.81^***^	0.85^***^
General Health	0.22	0.23	0.21	0.19	0.33^**^	0.24^*^
Vitality	0.45^***^	0.51^***^	0.42^***^	0.50^***^	0.61^***^	0.50^***^
Social Functioning	0.64^***^	0.63^***^	0.62^***^	0.72^***^	0.75^***^	0.68^***^
Role Emotional	0.53^***^	0.57^***^	0.53^***^	0.65^***^	0.73^***^	0.61^***^
Mental Health	0.47^***^	0.49^***^	0.45^***^	0.52^***^	0.64^***^	0.52^***^
PCS	0.80^***^	0.76^***^	0.79^***^	0.82^***^	0.63^***^	0.81^***^
MCS	0.56^***^	058^***^	0.53^***^	0.63^***^	0.74^***^	0.62^***^
QuickDash	−0.90^***^	−0.89^***^	−0.88^***^	−0.88^***^	−0.80^***^	−0.91^***^

*Abbreviations*: *WORC* Western Ontario Rotator Cuff index, *SF-36 PCS* SF-36 questionnaire Physical Component Summary, *SF-36 CS* SF-36 questionnaire Mental Component Summary, *QuickDash* an abridged version of the Disabilities of the Arm, Shoulder and Hand questionnaire

Statistically significant correlations ^*^ *p* < 0.05, *p* < 0,01^**^, *p* < 0,001^***^

**Fig. 1:**
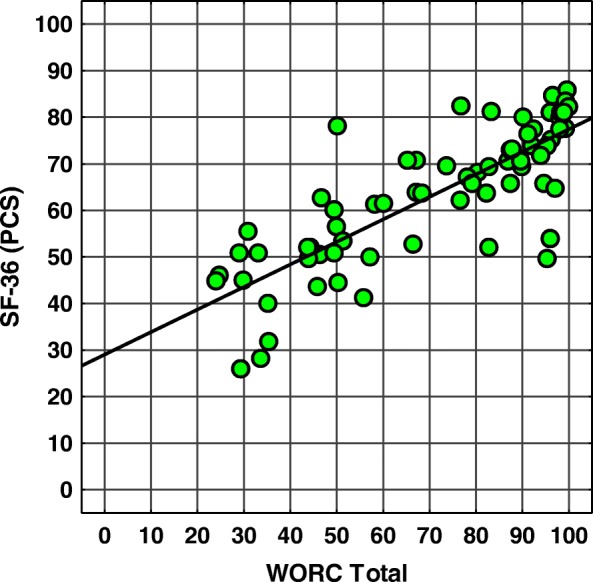
Scatter plot of WORC total scores vs. SF-36 PCS scores. Spearman’s correlation coefficient SCC = 0,81, *p* < 0,001. Abbreviations: *WORC* Western Ontario Rotator Cuff index, *SF-36 (PCS)* SF-36 questionnaire (Physical Component Summary)

**Fig. 2:**
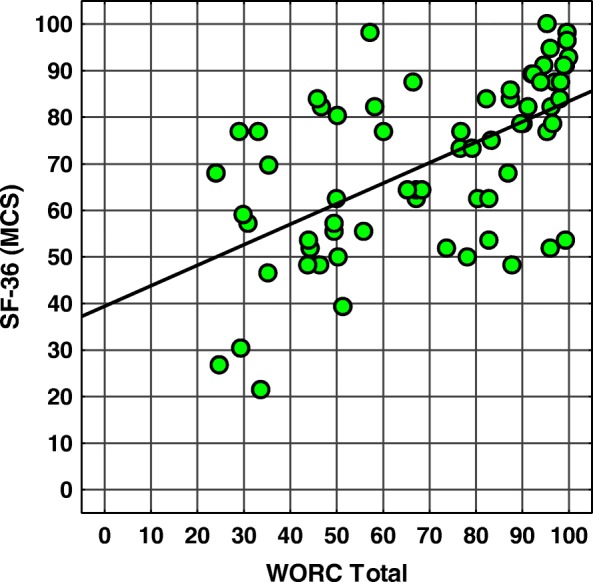
Scatter plot of WORC total scores vs. SF-36 MCS scores. Spearman’s correlation coefficient SCC = 0,62, *p* < 0,001. Abbreviations: *WORC* Western Ontario Rotator Cuff index, *SF-36 (MCS)* SF-36 questionnaire (Mental Component Summary)

**Fig. 3:**
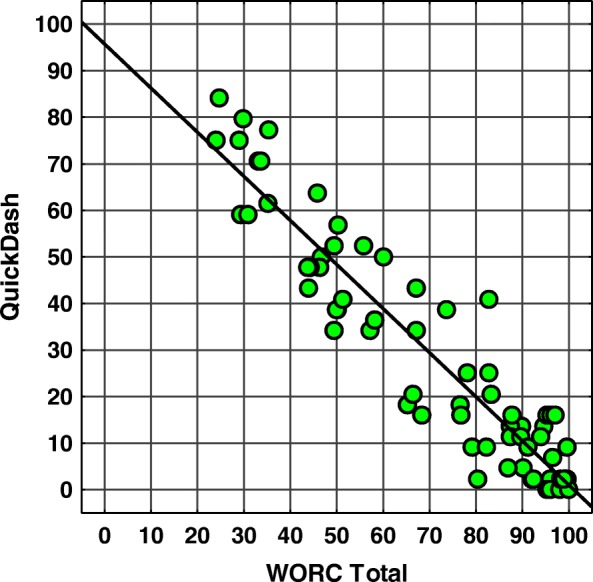
Scatter plot of WORC total scores vs. QuickDash. Spearman’s correlation coefficient SCC = − 0,91, *p* < 0,001. Abbreviations: *WORC* Western Ontario Rotator Cuff index, *QuickDash* an abridged version of the Disabilities of the Arm, Shoulder and Hand questionnaire

Figures 1, 2 and 3 show a scatter plots of WORC Total vs. PCS, WORC Total vs. MCS and WORC Total vs. QuickDash to illustrate the correlation between the scores.

#### Content validity

The floor effect does not appear in the WORC, SF-36 PCS and SF-36 MCS questionnaire, and the QuickDASH questionnaire is only marked in 5 people (7.2%). The ceiling effect is noted only in 1 person (1.4%) in the WORC questionnaire and SF-36 MCS, and in the other tools it does not occur. These results showed that all the outcome tools have similar floor and These results showed that all the outcome tools have similar floor and ceiling effects.

## Discussion

All of the hypothesis described in the methodology proved to be true. The WORC had very high test-retest reliability (Total ICC = 0.99, Domains 0.94–0.99). It had a high degree of internal consistency (Cronbach’s alpha for Total = 0.94, Domains = 0.92–0.95). Our validity comparisons also proved to be true with the strongest correlation between the Physical domain of the WORC and the SF-36 PCS. All correlations between the Total and the WORC domains and the QuickDash also proved to be strong as expected.

The standard methodology of translation, cultural adaptation and validation of research tools should be taken to ensure that the new language version developed is equivalent to the original version [21]. The use of official language versions of questionnaires or scales is necessary to preserve the comparability of research results carried out between researchers from the developing country as well as from various countries around the world [21, 37].

The WORC questionnaire was adapted to the Polish language due to the good psychometric properties of the original. It includes five domains of health as defined by the World Health Organization and collects information not covered by other research tools. The questionnaire can be used not only in the research setting but also in the clinical setting for monitoring an individual patient’s progress and for decision-making about treatment [9]. Bejer et al. [27] found the Polish WORC to be reliable and described the cultural and linguistic adaptation. The internal consistency of the Total WORC was assessed using Cronbach’s alpha and was found to be 0.94, with the range for individual items and domains from 0.88 to 0.95. This confirms good internal consistency. Any results lower than 0.70 may indicate a lack of correlation between the items in a scale, while higher might imply redundancy among the questions [33]. Our results are similar to the results of the authors of other the WORC validations. Cronbach’s alpha in the overall scale and subscales of the Chinese version of WORC is from 0.872 to 0.954 [26], Japanese version 0.78–0.95 [24], Danish version 0.91–0.97 [23], the Brazilian Portuguese version 0.88–0.97 [20], Swedish 0.89–0.97 [25]. Slightly lower values of the Cronbach’s alpha coefficients were obtained in the Turkish version 0.69–0.92 [19].

In our study the ICC for the different domains and for the total WORC ranged from 0.94 and 0.99. According to the literature guidelines, these results are acceptable and suitable for use in both group comparison studies and for evaluation of change in individuals [38]. Similar results were also obtained by the authors of other adaptations. In research from Norway, the ICC of the total WORC and individual domains ranged between 0,74 and 0.84 [21], in Turkey it ranged between 0.96 and 0.98, in Japan it ranged from 0.72 to 0.84 [19], in Denmark from 0.85–0.94 [23], in Sweden from 0.84 and 0.98 [25], in China from 0.82 and 0.96 [26] and in Brazil from 0.95 and 0.99 [20].

The Standard Error of the Means (SEM) associated with the total WORC was 1.62 points in our study, while Lopes et al. found the Brazilian WORC to be 3.0 points [20]. Our study indicates that clinicians can be confident that the total WORC score falls within 1.62 points (SEM) over a short time interval. We used the Minimal Detectable Change (MDC) to see if true change has occurred in an individual patient’s WORC total score. The MDC for the Total WORC was to 4.48 points (95% CI), while in the Brazilian study by Lopes et al. MDC was 7.1 points (90%CI) [16]. Our research indicates that a real change will occur in a patient if his result changes by more than 4.48 points. In Brazil, this change will occur with a slightly higher result - over 7.1 points [20].

We found that the Polish version of the WORC has good content validity. Floor and ceiling effects are considered to be present if more than 15% of the respondents achieved the lowest or highest possible score [33]. In the WORC questionnaire, there was a small and acceptable ceiling effect (1.4%). The other questionnaires tested (QuickDASH, SF-36 PCS and MCS) also presented a small ceiling and floor effect (max. 7.2%). In a Swedish study, Zhaeentan et al. stated that there were neither floor nor ceiling effects preoperatively but all instruments (WORC, WOOS, OSS and EQ-5D) had some ceiling effect postoperatively of approximately 10%. The EQ-5D had an unacceptably high ceiling effect of 32.3% while the specific health instruments were acceptable [25]. Similarly, Witte et al. found no floor or ceiling effects in the English version of the WORC [39].

Construct validity was assessed by the Spearman correlations coefficient between the WORC and the QuickDASH, a the specific tool to assess disability of the upper limb and the SF-36, a generic tool to assess the quality of life. As expected, correlations with the QuickDASH (0.80–0.91) are stronger than with the SF-36 (0.19–0.84). The WORC Total is moderately correlated with the SF-36 MCS - 0.62 and strongly correlated with the SF-36 PCS - 0.81. Weak correlations or lack of them only occurs with the SF-36 General Heath domain. Moderate or good correlations (0.47–0.79) were obtained between the physical subscales of the Chinese WORC and the OSS and the physical subscales of SF-36. The emotions subscale of the WORC and the mental subscales of SF-36 (0.52–0.71) correlate similarly [26]. We found similar relations in our study. Kirkley et al. found a moderate correlation between the original English WORC and other shoulder and upper extremity questionnaires: the American Shoulder and Elbow Surgeons Standardized Shoulder Assessment Form (ASES) (*r* = 0.68), the DASH (*r* = 0.63), the Constant Score (*r* = 0.63) and the University of California Los Angeles (UCLA) Shoulder Rating Scale (*r* = 0.48) [9]. The authors of subsequent validations confirm the above relationships. Between the Swedish version of the WORC and the WOOS there are strong correlations (0.97) [25]. The Japanese version of the WORC correlates more strongly with the DASH (*r* = 0.63–0.78) than with the SF-36 (*r* = − 0.24 to − 0.69) [24]. The Brazilian version also has marked strong correlations between the WORC and both the DASH and the UCLA Shoulder Rating Scale (*r* = − 0.86 and *r* = 0.80, respectively). Moderate correlations were found between the WORC and the SF-36 domains. The correlation was stronger with the SF-36 physical health summary score than with the mental health summary score [20].

### Limitations and future studies

The current study did not include the assessment of the responsiveness of the WORC and does not contain a control group. Future studies should be undertaken to assess the ability of WORC to detect Minimally Important Changes (MIC) over a period of time longer than 2 weeks and to assess the discriminatory power of the WORC.

## Conclusion

The Polish version of the WORC questionnaire can be considered a reliable and valid research tool with high internal consistency used to assess the quality of life in patients with arthroscopic rotator cuff repair. The psychometric properties of the Polish version of the WORC are comparable both with its original version as well as adaptations of WORC published in other countries.

## Abbreviations

$$ \overline{x} $$: (Mean)
%: Per cent
ASES: American Shoulder and Elbow Surgeons Standardized Shoulder Assessment Form
CI: Confidence Interval
DASH: Disabilities of the Arm, Shoulder and Hand Questionnaire
GRC: Global Rating of Change Scale
HRQOL: Health Related Quality of Life
ICC: Intraclass Correlation Coefficient
IST: Infraspinatus Tendon
LHBT: Long Head Biceps Tendon
MDC: Minimal Detectable Change
MIC: Minimally Important Changes
N: Number
QuickDash: Disabilities of Arm, Shoulder and Hand Questionnaire (an abridged version)
RC-QOL: Rotator Cuff Quality of Life Index
SCC: Spearman’s Correlation Coefficient
SEM: Standard Error of Measurement
SF-36 MCS: SF-36 Questionnaire - Mental Component Summary
SF-36 PCS: SF-36 Questionnaire - Physical Component Summary
SF-36 v. 2.0: Short Form-36 Questionnaire - version 2.0
SIQ: Shoulder Instability Questionnaire
SScapT: Subscapularis Tendon
SST: Supraspinatus Tendon
UCLA: University of California Los Angeles Shoulder Rating Scale
WOOS: Western Ontario Osteoarthritis of the Shoulder Index
WORC: Western Ontario Rotator Cuff Index
WOSI: Western Ontario Shoulder Instability Index
